# Calendar age and puberty-related development of regional gray matter volume and white matter tracts during adolescence

**DOI:** 10.1007/s00429-020-02208-1

**Published:** 2021-01-20

**Authors:** Ayaka Ando, Peter Parzer, Michael Kaess, Susanne Schell, Romy Henze, Stefan Delorme, Bram Stieltjes, Franz Resch, Romuald Brunner, Julian Koenig

**Affiliations:** 1 Section for Experimental Child and Adolescent Psychiatry, Department of Child and Adolescent Psychiatry, Centre for Psychosocial Medicine, University of Heidelberg, Blumenstr. 8, 69115 Heidelberg, Germany; 2 Clinic for Child and Adolescent Psychiatry, Centre for Psychosocial Medicine, University of Heidelberg, Heidelberg, Germany; 3 University Hospital of Child and Adolescent Psychiatry and Psychotherapy, University of Bern, Bern, Switzerland; 4 Section for Translational Child and Adolescent Psychiatry, Department of Child and Adolescent Psychiatry, Centre for Psychosocial Medicine, University of Heidelberg, Heidelberg, Germany; 5 Department of Psychiatry, Psychotherapy and Psychosomatics, Evangelisches Krankenhaus Königin Elisabeth Herzberge, Berlin, Germany; 6 Department of Psychology, Humboldt-Universität zu Berlin, Berlin, Germany; 7 Clinical Psychology and Psychotherapy, Freie Universität Berlin, Berlin, Germany; 8 Department of Radiology, German Cancer Research Centre, Heidelberg, Germany; 9 Department of Radiology and Nuclear Medicine, Universitätsspital Basel, Basel, Switzerland; 10 Clinic for Child and Adolescent Psychiatry, Psychosomatics and Psychotherapy, University of Regensburg, Regensburg, Germany

**Keywords:** Adolescent brain development, Puberty, Age, Magnetic resonance imaging (MRI), Gray matter volume, White matter tracts

## Abstract

**Background:**

Adolescence is a critical time for brain development. Findings from previous studies have been inconsistent, failing to distinguish the influence of pubertal status and aging on brain maturation. The current study sought to address these inconsistencies, addressing the trajectories of pubertal development and aging by longitudinally tracking structural brain development during adolescence.

**Methods:**

Two cohorts of healthy children were recruited (cohort 1: 9–10 years old; cohort 2: 12–13 years old at baseline). MRI data were acquired for gray matter volume and white matter tract measures. To determine whether age, pubertal status, both or their interaction best modelled longitudinal data, we compared four multi-level linear regression models to the null model (general brain growth indexed by total segmented volume) using Bayesian model selection.

**Results:**

Data were collected at baseline (*n* = 116), 12 months (*n* = 97) and 24 months (*n* = 84) after baseline. Findings demonstrated that the development of most regional gray matter volume, and white matter tract measures, were best modelled by age. Interestingly, precentral and paracentral regions of the cortex, as well as the accumbens demonstrated significant preference for the pubertal status model. None of the white matter tract measures were better modelled by pubertal status.

**Limitations:** The major limitation of this study is the two-cohort recruitment. Although this allowed a faster coverage of the age span, a complete per person trajectory over 6 years of development (9–15 years) could not be investigated.

**Conclusions:**

Comparing the impact of age and pubertal status on regional gray matter volume and white matter tract measures, we found age to best predict longitudinal changes. Further longitudinal studies investigating the differential influence of puberty status and age on brain development in more diverse samples are needed to replicate the present results and address mechanisms underlying norm-variants in brain development.

**Supplementary Information:**

The online version contains supplementary material available at 10.1007/s00429-020-02208-1.

## Introduction

Adolescence is a critical period of brain development. Structural and functional interconnectivity of brain regions form a reliably and accurately functioning network to efficiently process information and behavioural output. The development of the brain during adolescence has predominantly been studied in relation to age (Andersen 2003; Blakemore 2012; Giedd and Rapoport 2010; Lenroot and Giedd 2006; Mills and Tamnes 2014). During normative gray matter development, a reduction in general cortical volume with increasing age has been consistently reported (Ducharme et al. 2016; Mills et al. 2016; Vijayakumar et al. 2016; Wierenga et al. 2014). However, more recently, studies have highlighted that the influence of pubertal maturation—not only age—on brain growth is crucial in disentangling the underlying physiological mechanisms (Kaczkurkin et al. 2018).

When considering the effect of age and pubertal status on brain development, it is important to note that the age in which biological changes occur in association with puberty varies across individuals. Therefore, age and pubertal status need to be considered as partially distinctive measures. For example, variation in puberty onset can differ by up to 4–5 years (Parent et al. 2003). More recent studies have hence examined the effects of pubertal status on brain development, which has also been recently reviewed (Mills and Tamnes 2014; Vijayakumar et al. 2018). Existing findings suggest that although pubertal status is associated with overall brain development and maturation, particularly in the frontal cortex, findings are inconsistent potentially due to disparities in study designs and measures used. Moreover, previous studies investigating white matter tract development in association with pubertal status are scarce. Consequently, it is still unclear how both age and/or pubertal development are associated with individual trajectories of specific regional brain development.

The present study sought to investigate individual trajectories in brain development comparing the influence of age and pubertal status with a longitudinal design of repeated within-subject measurements during adolescence. More specifically, we aimed to describe longitudinal changes in regional gray matter volume and white matter tract measures modelled by normative physical growth, age, or pubertal status in healthy adolescents tracked over 3 years.

## Methods

### Study design and recruitment

The study was approved by the human research ethics committee of the Medical Faculty at Heidelberg University, Germany (study ID: S-604/2011). All participants and at least one legal guardian signed written informed consent prior to inclusion in the study. Recruitment took place in Heidelberg, Germany in 2014 and 2015. Children were recruited from the general public using letters sent out to households (*n* = 2398) with children within the age range of interest, identified by contacting the citizens registration office at Heidelberg City Council, Germany.

Participants with a history of psychiatric diagnosis or treatment, developmental disorders, premature birth (birth weight below 2000 g and/or birth before 36th week of pregnancy), intellectual impairment (i.e., IQ < 80), poor knowledge of the German language, or those reporting endocrine disorders were excluded. Adolescents were also excluded if either of their parents had a history of psychiatric diagnosis or treatment. Exclusion criteria also included MRI safety regulations, in which participants reporting claustrophobia, those with metal implants, a history of brain injury, or vascular/neurological diseases (i.e., pathology that may influence brain function) were excluded.

After a screening interview, participants subsequently underwent a structured assessment (*detailed below*). Participants who agreed to undergo MRI scanning were invited to the Division of Radiology at the German Cancer Research Center, Germany, for a second appointment (*detailed below*). To capture 6 years of normative brain development, two cohorts of healthy children and adolescents were recruited: a) cohort 1, which consisted of 9–10 years old and b) cohort 2, which consisted of 12–13 years old at baseline. These children and adolescents were subsequently followed-up every year until 2 years after the initial scan, making a total of three time-points. Further details on participant flow and reasons for the exclusion of subjects are provided elsewhere (Mürner-Lavanchy et al. 2020).

### Structured assessments

The first assessment consisted of interviews to collect data on demographics, pubertal status using the *Pubertal Development Scale* (Petersen et al. 1988). The PDS is a self-report measure composed of 5 items. Three general items (for boys and girls) assess growth spurts, changes in body hair, and skin. Each of these is rated on a 5-point scale: “not yet started”, “barely started”, “definitely started”, “seems complete”, and “I don’t know” (treated as missing). Girls are further asked to indicate breast growth and onset of menstruation. Boys are asked to indicate changes in voice and facial hair growth. Puberty status is indicated on a 5-point scale (*prepubertal*, *early pubertal*, *mid pubertal*, *late pubertal*, and *post pubertal*). Intelligence level was assessed using the *General Ability Index* (GAI; (Raiford et al. 2005), which is a compressed measure of general intellectual ability measured by the German version of the *Wechsler Intelligence Scale for Children, fourth edition* (Petermann and Petermann 2008; Wechsler 2003). Psychiatric health of the participants was confirmed using the German version of the *Mini-International Neuropsychiatric Interview for Children and Adolescents* (M.I.N.I- KID 6.0) (Sheehan et al. 2010). All interviews were performed by trained clinicians in the field of child and adolescent psychiatry.

### Neuroimaging

MRI data were acquired using a Siemens 3 T Biograph mMR with a 16-channel head coil with a total acquisition time of 45 min. Anatomical T1-weighted images were acquired in the sagittal plane (192 slices, 1 mm slice thickness, 1 × 1 mm2 in-plane resolution, echo time (TE) = 2.98 ms, repetition time (TR) = 2300 ms, and flip angle = 9). A whole-brain single-shot spin-echo echo-planar imaging sequence was used to obtain the DTI images using the following parameters in axial plane: 50 slices, 64 directions, voxel size = 2.5 mm isotropic, TE = 112 ms, TR = 12,100 ms, FOV = 240 mm, matrix size = 96 × 96, and b values at 0, 1000 and 3000 s/mm). A GeneRalised Autocalibrating Partially Parallel Acquisition (GRAPPA) factor 2 was used.

Automated methods were used for calculating both gray matter volume and white matter tract measures. Each T1 and DTI image was visually checked for quality assurance by trained researchers before automated processing. Participants with abnormal scan reports or with movements of more than 2 mm were discarded from further analyses. Subsequent to analyses, outputs were overlapped onto native T1 image for additional quality assurance. For structural segmentation to calculate gray matter volumes, FreeSurfer version 6.0 was used to segment T1-weighted images (Fischl 2012). Details of FreeSurfer segmentation are described in previous papers by Fischl et al. (Fischl et al. 2002, 2004). Outputs from regions listed in the Desikan-Killiany-Tourville atlas were used (Klein and Tourville 2012). For calculating fractional anisotropy (FA) and mean diffusivity (MD) measures of the white matter tracts, DTI data were processed using TRACULA (TRActs Constrained by UnderLying Anatomy, FreeSurfer). This automated method includes pre-processing such as standard methods for image distortion correction of eddy currents and subject movement (Andersson and Sotiropoulos 2016) and B0 field inhomogeneity, intra-subject and inter-subject registration, and tensor fitting as detailed in previous papers (Yendiki 2011; Yendiki et al. 2014). As part of the FreeSurfer suite, TRACULA uses not only anatomical priors derived from an atlas, but also the cortical parcellation and subcortical segmentations derived from native space of each subject in FreeSurfer, allowing individual variations across subjects while still establishing the same tracts for comparison (Jbabdi et al. 2007).

### Statistical analyses

A total of five multi-level linear regression models were calculated for each outcome of interest and compared to a null model (M_0_) using Bayesian model selection. The null model only included total segmented volume as fixed factor to predict regional brain volume or the white matter tract measure of interest (dependent variables). The four models of interest were composed as follows: Model 1 (M_1_): fixed factor age (exact age at the time of the scan including 6 decimals); Model 2 (M_2_): fixed factor puberty status; Model 3 (M_3_): both age and puberty status as fixed factors; Model 4 (M_4_): age and pubertal status main and interaction effects. All models included sex as a covariate and subject as random effect. Total segmented volume was added as additional covariate for all models of interest (M_1_ to M_4_) to control for global effects of brain size (e.g., O’Brien et al. 2011; Peelle et al. 2012 Each model (M_1_ to M_4_) was compared to the null model (M_0_) using Bayesian Information Criterion (BIC) differences as an estimation of the Bayes Factor (BF). Interpretation of the BF was based on the proposed convention by (Raftery et al. 1995). Beta values for fixed factors correcting for covariates were calculated to illustrate the direction in developmental trajectories based on methods outlined by Hox (1995). For the sake of completeness, we also report on global brain effects All statistical analyses were performed using Stata version 15 (StataCorp 2017).

## Results

### Sample characteristics

After screening interested participants (*n* = 228), *n* = 125 met the inclusion criteria. Data were collected at three time-points at baseline (time-point 1; interview: *n* = 125; MRI: *n* = 114), 12 months from baseline (time-point 2; interview: *n* = 111; MRI: *n* = 94), and 24 months from baseline (time-point 3; interview: *n* = 106; MRI: *n* = 80). Reasons for drop-outs throughout the three time-points included claustrophobia during MRI scan, which was otherwise unknown prior to the MRI scanning session (*n* = 2; 1.6%), termination of scan due to headache, dizziness of feeling sick (*n* = 7; 5.6%), withdrawal due to loss of interest (*n* = 9; 7.2%), no show for appointments (*n* = 6; 4.8%), or when the participants and their parent were no longer contactable (*n* = 2; 1.6%). The numbers of participants’ MRI data specified in the results demonstrate the final number of MRI data used in the analyses, which excludes images that contained large movement or other artefacts associated with image acquisition (*n* = 6; 4.8%), or artefacts caused by dental braces (*n* = 13; 10.4%). Summary of demographic information on the cohorts is given in Table 1.

**Table 1:** Demographic information of participants who were included in the MRI analysis.

		Cohort 1^a^	Cohort 2^b^
Baseline	*n* (females)	55 (26)	59 (30)
	Mean age (SD)	9.61 (0.34) [8.85–10.29]	12.62 (0.32) [11.86–13.03]
	Puberty status (SD)	1.4 (0.60) [1–3]	2.81 (0.99) [1–4]
	Total segmented volume^c^ (SD)	1179287 (107546.2) [947801–1464491]	1191869 (108286.7) [975723–1420689]
	IQ (HAWIK-GAI)	119.49 (13.45) [92–148]	117.92 (11.64) [87–146]
12 months	*n* (females)	48 (22)	46 (21)
	Mean age (SD)	10.81 (0.38) [9.93–11.39]	13.85 (0.43) [12.94–14.86]
	Puberty status (SD)	1.75 (0.76) [1–3]	3.28 (0.89) [1–5]
	Total segmented volume^c^ (SD)	1196196 (106075.1) [938994–1439917]	1191269 (103432.8) [986087–1414269]
	Days since baseline (SD)	413.53 (65.61) [279–579]	415.33 (62.91) [333–611]
24 months	*n* (females)	41 (17)	39 (17)
	Mean age (SD)	11.80 (0.38) [10.97–12.50]	14.86 (0.41) [14.21–16.01]
	Puberty status (SD)	2.10 (0.80) [1–3]	3.51 (0.85) [1–5]
	Total segmented volume^c^ (SD)	1187069 (99999.5) [988972–1393745]	1193478 (113822.7) [988529–462798]
	Days since 12 months (SD	342.90 (75.70) [210–676]	358.54 (97.06) [214–737]

*SD* standard deviation; range: in square brackets; *IQ* intelligence quotient; HAWIK-GAI Hamburg-Wechsler Intelligenztest für Kinder-General Ability Index.

^a^Cohort 1, 9–10 years old at baseline

^b^Cohort 2, 12–13 years old at baseline

^c^Total segmented volume presented in mm^3^

### Model comparisons

**Fig. 1:**
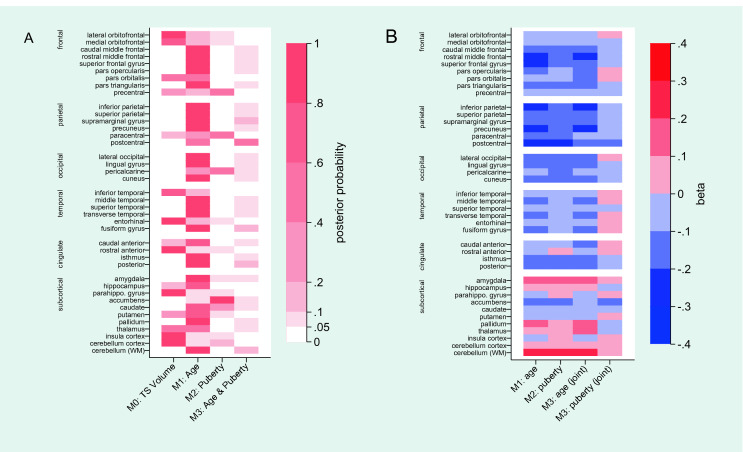
Preferred model in predicting regional gray matter volume development; illustrated is the posterior probability of each model (a) and Beta values for fixed factors of each model (M_1_, M_2_, and M_3_) for predicting regional gray matter volume adjusted for covariates

When testing for global effects on total segmented volume, we found no superior model fit for age (M_1_: BIC: − 5.64; BF: 0.06; Prob: 0.05); pubertal status (M_2_: BIC: − 5.35; BF: 0.07; Prob: 0.06); both main effects (M_3_: BIC: − 10.93; BF: 0.00; Prob: 0.00); or their interaction (M_4_: BIC: − 13.04; BF: 0.00; Prob: 0.00) against a null model (sex only; M_0_: BIC: 0.00; BF: 1.00; Prob: 0.88). For region-specific models, model 4 (M_4_: age and pubertal status interaction as fixed factor) was not the preferred model for any of the model comparisons, as indicated by model fit comparisons. Therefore, M_4_ was removed from all subsequent model comparisons. Model comparison for regional gray matter volumes demonstrated that model preference was distributed throughout the brain. Cortical and subcortical regions that were tested, as well as their preferred model are reported in Fig. 1a, with full reports on BIC difference, the BF, and the respective probability of comparisons in the *Supplementary Material*. Cortices that showed the null model (M_0_) as the best fitting model included cerebellum, lateral and medial orbitofrontal, pars orbitalis, entorhinal, interior temporal, rostra anterior cingulate cortices, and subcortical regions such as the insula and the parahippocampal gyrus. As illustrated in Fig. 1a, the preferred model and its representation on the brain, most regional gray matter volumes were best modelled by age (M_1_). These included regions of the frontal cortex, parietal cortex, occipital cortex, temporal cortex, the cingulate cortex and subcortically the amygdala, hippocampus, caudate, pallidum, and putamen. Regional gray matter volumes that were best modelled by pubertal status (M_2_) included the precentral region, paracentral lobule, pericalcarine sulcus, and subcortically the accumbens. The postcentral region was the only gray matter region best modelled with the addition of both age and pubertal status as fixed factors (M_3_). Graphical representations of the preferred model predicting gray matter development are provided in Fig. 1a. Beta values for the fixed factors of each model are presented in Fig. 1b.

**Fig. 2:**
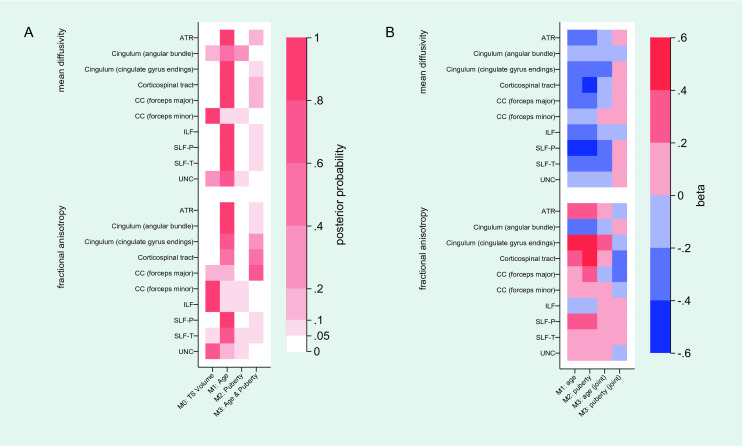
Preferred model in predicting fractional anisotropy and mean diffusivity measures of white matter tracts; illustrated is the (**A**) posterior probability of each model predicting mean diffusivity and fractional anisotropy measures of white matter tracts. Furthermore, (**B**) beta values adjusting for covariates for fixed factors of each model (M_1_, M_2_, and M_3_) for predicting mean diffusivity and fractional anisotropy measures of white matter tracts are illustrated. *Abbreviations*; MD: mean diffusivity; FA: fractional anisotropy; ATR: anterior thalamic radiation; ILF: inferior longitudinal fasciculus; SLF-P: superior longitudinal fasciculus-parietal endings; SLF-T: superior longitudinal fasciculus-temporal endings; UCF: uncinate fasciculus; CC: corpus callosum

FA and MD of the white matter tracts tested and the preferred model for each measure is indicated in Fig. 2. Full reporting on BIC difference, the BF and the respective probability of the comparisons is provided in the *Supplementary Material*. The corpus callosum—forceps minor—as well as the FA of the inferior longitudinal fasciculus and uncinate fasciculus were best modelled by general brain size indicated by total segmented volume (M_0_). Once more, age was the preferred model (M_1_) for a majority of the tracts including MD and FA of the anterior thalamic radiations, both parietal and temporal endings of the superior longitudinal fasciculus, cingulum—angular bundle, cingulum—cingulate gyrus endings, as well as MD of the inferior longitudinal fasciculus, uncinate fasciculus, corpus callosum—forceps major and cortico-spinal tract. FA of the corpus callosum—forceps major and the cortico-spinal tract were the only white matter tract measures that were best modelled with the addition of both age and pubertal status as fixed factors (M_3_). Pubertal status as fixed factor (M_2_) was not the preferred model predicting FA and MD measures from any of the white matter tracts under investigation. Graphical representations of the preferred model predicting white matter tract development are provided in Fig. 2a. Beta values for the fixed factors of each model are also presented in Fig. 2b.

## Discussion

The present study compared the influence of age and pubertal status on the longitudinal trajectory of structural brain development in adolescents. Following an exploratory approach, we addressed the impact of age and puberty status on gray matter volume in 39 regions of interest and 10 white matter tracts. Given the plenitude of statistical tests, we relied on Bayesian statistics to minimize potential error associated with multiple testing under a frequentist framework. Analyses demonstrated that the development of gray matter volume and white matter tracts during adolescence is overall best predicted by age. Interestingly, Bayesian model selection revealed that gray matter volume development in precentral and paracentral regions of the cortex, as well as subcortical regions including the accumbens are better predicted by pubertal status. None of the white matter tract measures were better modelled by pubertal status, but FA values of the corpus callosum (forceps major) and cortico-spinal tract were better predicted by the addition of both age and pubertal status as fixed factors in a joint model.

Previous studies investigating the effects of pubertal status on gray matter development have shown negative associations between global gray matter volume and pubertal status, as well as gonadal hormone levels (Bramen et al. 2011; Paus et al. 2010; Peper, Brouwer, et al. 2009a, b; Pfefferbaum et al. 2016). Other studies have shown no significant relationship between gray matter volume and puberty-associated changes (Brouwer et al. 2015). The most consistent regional finding to date has been the association between pubertal status and the development of frontal and temporal lobes, as well as the anterior cingulate cortex in both cross-sectional and longitudinal studies (Bramen et al. 2012; Herting et al. 2015; Hu et al. 2013; Koolschijn et al. 2014; Nguyen et al. 2013a, b; Peper, Schnack et al. 2009a, b; Pfefferbaum et al. 2016). Interestingly, however, the results observed in the current study of longitudinal development showed that most of these regions are, however, better modelled by age rather than pubertal status, with most regions showing a negative association indicating a general decrease in gray matter volume with increasing age.

Additionally, the amygdala and hippocampus have consistently been highlighted as subcortical regions of interests when investigating brain development during puberty due to the dense population of sex steroid hormone receptors in these regions (Abdelgadir et al. 1999). The current study investigated not only the amygdala and hippocampus, but a wider network of subcortical regions including the parahippocampal gyrus, accumbens, caudate, putamen, pallidum, and thalamus. Amygdala volume has been shown to have a negative association with female pubertal status, but a positive association with male pubertal status (Blanton et al. 2012; Hu et al. 2013). Hippocampus volume has also revealed a negative association with pubertal status (Blanton et al. 2012; Neufang et al. 2009), but with two studies showing sexual dimorphic patterns (Bramen et al. 2012; Hu et al. 2013). Interestingly, the amygdala and hippocampus did not show preference for pubertal status in the current study but rather the accumbens. In animal studies, the accumbens has been shown to be related to the dopaminergic pathways associated with pubertal hormonal changes, such that there is increased motivation for reward seeking behaviour including sexual behaviour (Sato et al. 2008). However, the distinct association of pubertal alteration in the accumbens in humans is still unclear and further longitudinal studies building on the present findings are needed.

Moreover, our analyses showed that neither FA nor MD of any of the tracts that were tested showed significant preference for the pubertal status model. However, previous research investigating white matter tract development in association with pubertal status has been scarce. While some studies implicate a predominantly positive association between pubertal status and FA (Herting et al. 2012; Menzies et al. 2015), only a few studies have observed any association with pubertal status and MD (Menzies et al. 2015). The few studies that investigated such association found similarities to findings on gray matter volume development, where cortico–cortico and cortico-subcortical tracts that are associated with the frontal and temporal lobes showed the most consistent associations with pubertal status (Herting et al. 2012; Menzies et al. 2015). In the current study, however, cortico-spinal and hemispheric connections were associated with age and pubertal status, where FA of both of these tracts were negatively associated with age and pubertal status. Most interestingly, beta values indicated that when both age and pubertal status are added as fixed factor in a joint model, FA has a positive association with age and a negative association with pubertal status. This finding highlights a potential role of cortico-spinal and hemispheric connections in studying norm-variants in delayed or premature pubertal development. Additional studies in more diverse samples are needed to ascertain and disentangle the intertwining influences of age and pubertal status in these regions of interest.

Inconsistencies seen with previous studies could be influenced by the various measures of pubertal status used. For example, there are two prominent systems for measuring pubertal status: the *Tanner Stage* (Tanner, 1692), and the PDS (Petersen et al. 1988). The two measures are not correlated, and therefore, they are potentially capturing differential aspects of pubertal development. The current study used the PDS (Petersen et al. 1988), and due to its self-report format, it could be considered as one of the limitations of the study, potentially introducing bias (Shirtcliff et al. 2009). Although the study design could improve by the addition of physical examination, we opted out of this option due to its intrusiveness. Another reason for inconsistent associations seen with brain development and puberty could be due to some studies using hormone measures as an indicator of pubertal development. Although hormones are a direct measure of pubertal maturation, there is unfortunately a wide range of variability in hormone levels during adolescence and its associated pubertal stages of development (Dorn et al. 2004; Dorn and Biro 2011). For example, hormone levels vary according to the time of day the sample is obtained, the menstrual cycle in females, diet, stress, and other environmental factors. Furthermore, differential collection and analysis methods, such as saliva, blood, and urine samples, have also produced varying results (Vesper et al. 2014). Measuring hormone levels from hair could provide a more stable measure in this regard.

One major pitfall of the present study is the two-cohort design that was applied to facilitate the recruitment process. Although this allowed a faster collection of data with a larger age range, a complete within-subject trajectory over 6 years could not be investigated. Therefore, we were only able to investigate the trajectory from three time-points for two cohorts rather than six time-points for the entire sample. The age of data collection is a crucial aspect for a study on longitudinal developmental. Past neuroimaging studies investigating gray matter development have reported widespread alterations in the cortex during adolescence. For example, an inverted U-shaped developmental pattern, peaking at various ages in different cortices, has been reported from studies using the National Institute of Mental Health Child Psychiatry dataset in males and females aged between 4 and 25 years (Giedd et al. 1999; Lenroot and Giedd 2006; Raznahan et al. 2014). However, other studies report a linear decrease in total gray matter volume across late childhood-to-adulthood, where the greatest proportion and highest rate of decrease in gray matter volume occurred in participants in their teens and no significant change was seen in participants aged between 22 and 32 years (Lebel and Beaulieu 2011; Tamnes et al. 2013; Wierenga et al. 2014). Another study showed stable volumes of gray matter up to 10 years old, and then, a decrease was observed between ages 10 and 20 (Aubert-Broche et al. 2013). These heterogeneous findings indicate that although there may not be a clear age of “peak” of gray matter volume, it reaches its maximum during late childhood and decreases throughout adolescence to adulthood, where volume loss decelerates. The current three time-points from ages 9 and 12 may not have best captured the peak of development. One advantage of the present study is that an automated method has been used to calculate both volume and white matter tract information, allowing fast, reliable, and easily reproducible results, which also reduces the risk of manual error or bias depending on the researcher. Moreover, the outputs from volumetric comparisons were used to compute white matter tract information, allowing the tract calculation to be completed in native space. However, given the focus on gray matter volume and white matter tracts, we did not assess other brain structural variables of potential interest such as surface area and cortical thickness. Finally, our sample represents a high-function group of individuals, as further reflected in the high average IQ scores, way beyond what would be expected for the norm. While likely linked to specifics in the regional catchment area, it is not clear how the present findings generalize to samples from a diverse educational or socioeconomic background.

To summarize, the present study highlights some regions of interest, which—alongside the majority of brain regions that are best predicted by age in their development—are driven in their development across adolescence by pubertal status. Further longitudinal studies are needed to replicate the findings in more diverse and larger samples across the pubertal age span. These studies could be improved by incorporating gonadal hormone measures in combination with physical examinations of pubertal stats. Furthermore, the addition of functional MRI and measures of socioenvironmental factors would aid our understanding on the physical development and associated changes in functional activity and connectivity.

## Electronic supplementary material

Below is the link to the electronic supplementary material.

(PDF 120 kb)

(PDF 259 kb)

(PDF 338 kb)

(PDF 689 kb)

(PDF 1491 kb)

(PDF 1493 kb)

(PDF 645 kb)

(PDF 1493 kb)

(PDF 69 kb)

(PDF 578 kb)

## Data Availability

The datasets generated during and/or analysed during the current study are available from the corresponding author on reasonable request.
