# Supplementary material for: Calendar age and puberty-related development of regional gray matter volume and white matter tracts during adolescence
Source: Brain Struct Funct. 2021 Jan 20;226(3):927–37. doi: 10.1007/s00429-020-02208-1 (PMC7981330; doi:10.1007/s00429-020-02208-1)
Supplement: Supplementary file 6 — (PDF 1493 kb) [file 429_2020_2208_MOESM6_ESM.pdf]

SPRINGER NATURE

Disclosure of potential conflicts of interest

Authors must disclose all relationships or interests that could have direct or potential influence or impart bias on the work. Although an author may not feel there is any conflict, disclosure of all relationships and interests provides a more complete and transparent process, leading to an accurate and objective assessment of the work. Awareness of real or perceived conflicts of interest is a perspective to which the readers are entitled. This is not meant to imply that a financial relationship with an organization that sponsored the research or compensation received for consultancy work is inappropriate. For examples of potential conflicts of interests *that are directly or indirectly related to the research please* visit:
http://www.springer.com/gp/authors-editors/journal-author/journal-author-helpdesk/publishing-ethics/14214

All authors of papers submitted to Brain Structure and Function
[*include name of journal*] must complete this form and disclose any real or perceived conflict of interest.

Please complete one form per author. The corresponding author collects the conflict of interest disclosure forms from all authors. The corresponding author will include a summary statement that reflects what is recorded in the potential conflict of interest disclosure form(s). Please check the Instructions for Authors where to put the statement which may be different dependent on the type of peer review used for the journal. Please note that you cannot save the form once completed. Please print upon completion, sign, and scan to keep a copy for your files.
The corresponding author should be prepared to send potential conflict of interest disclosure form if requested during peer review or after publication on behalf of all authors (if applicable).

[X] I have no potential conflict of interest.

Category of disclosure	Description of Interest/Arrangement

Article title Age and Puberty-Related Development of Regional Gray Matter Volume and White Matter Tracts During Adolescence

Manuscript No. (if you know it) ____________________

Author name Julian Koenig

Are you the corresponding author? [X] Yes [ ] No

Herewith I confirm that the information provided is accurate.

Author signature [signature] Date 08.11.2019
